# Evidence for accelerated aging in mammary epithelia of women carrying germline *BRCA1* or *BRCA2* mutations

**DOI:** 10.1038/s43587-021-00104-9

**Published:** 2021-09-14

**Authors:** Sundus F. Shalabi, Masaru Miyano, Rosalyn W. Sayaman, Jennifer C. Lopez, Tiina A. Jokela, Michael E. Todhunter, Stefan Hinz, James C. Garbe, Martha R. Stampfer, Kai Kessenbrock, Victoria E. Seewaldt, Mark A. LaBarge

**Affiliations:** 1 Department of Population Sciences, Beckman Research Institute, City of Hope, Duarte, CA, USA.; 2 Irell and Manella Graduate School of Biological Sciences, City of Hope, Duarte, CA, USA.; 3 Medical Research Center, Al-Quds University, Jerusalem, Palestine.; 4 Cancer Metabolism Training Program, City of Hope, Duarte, CA, USA.; 5 Department of Laboratory Medicine, Helen Diller Family Comprehensive Cancer Center, University of California, San Francisco, CA, USA.; 6 Biological Systems and Engineering Division, Lawrence Berkeley National Laboratory, Berkeley, CA, USA.; 7 Biological Chemistry Department, University of California, Irvine, CA, USA.; 8 Center for Cancer and Aging, City of Hope, Duarte, CA, USA.; 9 Center for Cancer Biomarkers Research, University of Bergen, Bergen, Norway.

## Abstract

During aging in the human mammary gland, luminal epithelial cells lose lineage fidelity by expressing markers normally expressed in myoepithelial cells. We hypothesize that loss of lineage fidelity is a general manifestation of epithelia that are susceptible to cancer initiation. In the present study, we show that histologically normal breast tissue from younger women who are susceptible to breast cancer, as a result of harboring a germline mutation in *BRCA1*, *BRCA2* or *PALB2* genes, exhibits hallmarks of accelerated aging. These include proportionately increased luminal epithelial cells that acquired myoepithelial markers, decreased proportions of myoepithelial cells and a basal differentiation bias or failure of differentiation of cKit^+^ progenitors. High-risk luminal and myoepithelial cells are transcriptionally enriched for genes of the opposite lineage, inflammatory- and cancer-related pathways. We have identified breast-aging hallmarks that reflect a convergent biology of cancer susceptibility, regardless of the specific underlying genetic or age-dependent risk or the associated breast cancer subtype.

Aging is the greatest risk factor for sporadic breast cancers^1^. However, despite the striking relationship between aging and cancer susceptibility, only one in eight women in any age group in the USA ultimately develops breast cancer. Most of these breast cancers are sporadic with idiopathic origins. Only 5–10% of breast cancers are due to one of a handful of germline mutations in genes such as *BRCA1*, *BRCA2*, *CHEK2*, *ATM*, *TP53* and *PALB*2. Women who carry these germline mutations are not only diagnosed more frequently with breast cancer but also are diagnosed at an earlier age^2^, for example, *BRCA1* mutation (*BRCA1*^mut^) carriers are estimated to have more than a 70% lifetime risk of a breast cancer diagnosis^2^. As a majority of these germline mutations are in genes that encode key DNA damage-repair proteins, we speculated that random physiochemical damages may accumulate more frequently and thus lead to an acceleration of biological age and commensurate aging phenotypes^3^.

The mammary gland is a bilayered epithelium with an inner layer of keratin 19 (KRT19)-expressing secretory luminal epithelial cells that is surrounded by an outer layer of contractile KRT14-expressing myoepithelial cells. Myoepithelial cells are epithelial cells that are considered to have basal properties and thought to be tumor suppressive^4,5^. This bilayered epithelium is surrounded by a basement membrane that separates the epithelial compartment from the adipose-rich stromal compartment. With age, the relative amount of adipose tissue in the stroma increases and connective tissue decreases^6^. In the epithelia, luminal cell proportions increase, myoepithelial cell proportions decrease and tyrosine kinase receptor cKit-expressing progenitors with a basal differentiation bias accumulate^6,7^. A striking age-dependent change in the luminal epithelia is the acquired expression of myoepithelial proteins such as the intermediate filament KRT14 (refs.^6–8^). We defined this age-dependent state as loss of lineage fidelity, in which luminal epithelia gain some characteristics of the myoepithelia while still retaining hallmark characteristics of luminal epithelial cells^8^. The phenotypic changes that occur in the luminal epithelia merit careful attention because cKit progenitors and more mature luminal cells are the putative breast cancer cells of origin^6,9–12^. Whether loss of fidelity is explicitly related to aging or more broadly related to cancer susceptibility is not known.

Epithelial plasticity is the ability of cells to transition between different metastable states by accessing the epithelial-to-mesenchymal transition (EMT) and stem-cell-related gene programs^13^. Epithelial plasticity is important in development but is also coopted by cancers. Epithelial plasticity is a prominent feature of basal-like breast cancers, which are primarily triple negative^14,15^. Basal-like breast cancers commonly arise in high-risk women carrying *BRCA1* mutations, and are known for their aggressiveness and resistance to chemotherapy^16,17^. This aggressive behavior has been attributed to increased plasticity and the ability to transition across progenitor, basal and luminal states^14^. We speculate that the age-dependent loss of lineage fidelity in luminal cells is a form of epithelial plasticity, and their acquisition of basal features is a step toward increased cancer susceptibility. Age is an important risk factor for breast cancer in the average-risk (AR) population, whereas carriers of certain germline gene variants are decidedly high risk even when they are premenopausal, and considered to be chronologically young. We hypothesized that loss of lineage fidelity in luminal epithelia is a biological emergent property of mammary epithelia that is susceptible to cancer initiation and is accelerated in high-risk (HR) women.

To determine whether the breast epithelial changes that we previously identified as age dependent are associated with breast cancer susceptibility due to predisposing mutations, we examined pathologically normal breast tissue from prophylactic mastectomies of women harboring germline mutations in breast cancer-susceptibility genes. Compared with AR controls, epithelia from clinically verified, germline, HR breast tissue exhibited expansion of luminal cells that expressed KRT14. Differentiation assays showed that cKit-enriched (cKit^+^) progenitors from HR epithelia had a basal bias irrespective of the specific germline mutation. Transcriptionally, HR luminal and myoepithelial cells are distinguishable from AR cells based on enrichment for aging, inflammatory and senescence gene signatures. The enrichment for some distinct gene signatures among the HR epithelia, based on the distinct underlying germline mutations, suggested that the cells produce mutation-specific microenvironments. We propose that predisposing germline mutations accelerate aging processes in mammary epithelia, resulting in compositional changes that reduce the ability of the tissue to suppress cancer initiation and increase the pool of cancer cells of origin.

## Results

### Definition of risk status and age groups.

Women were defined as high risk if they had a germline mutation that greatly increases lifetime risk of a breast cancer diagnosis including *BRCA1*, *BRCA2* and *PALB2* (refs.^2,18^). The age distribution of HR samples was from 24 years to 59 years, mean = 45.5 years (median = 48 years; Supplementary Table 1). Women were defined as AR if they did not harbor predisposing genetic mutations. For the present study, samples were designated as ‘younger’ if they were collected from women aged ≤35 years, ‘older’ if they were aged ≥55 years and ‘middle aged’ from women aged 36–54 years.

### HR luminal epithelia coexpress KRT14 and KRT19.

**Fig. 1 |:**
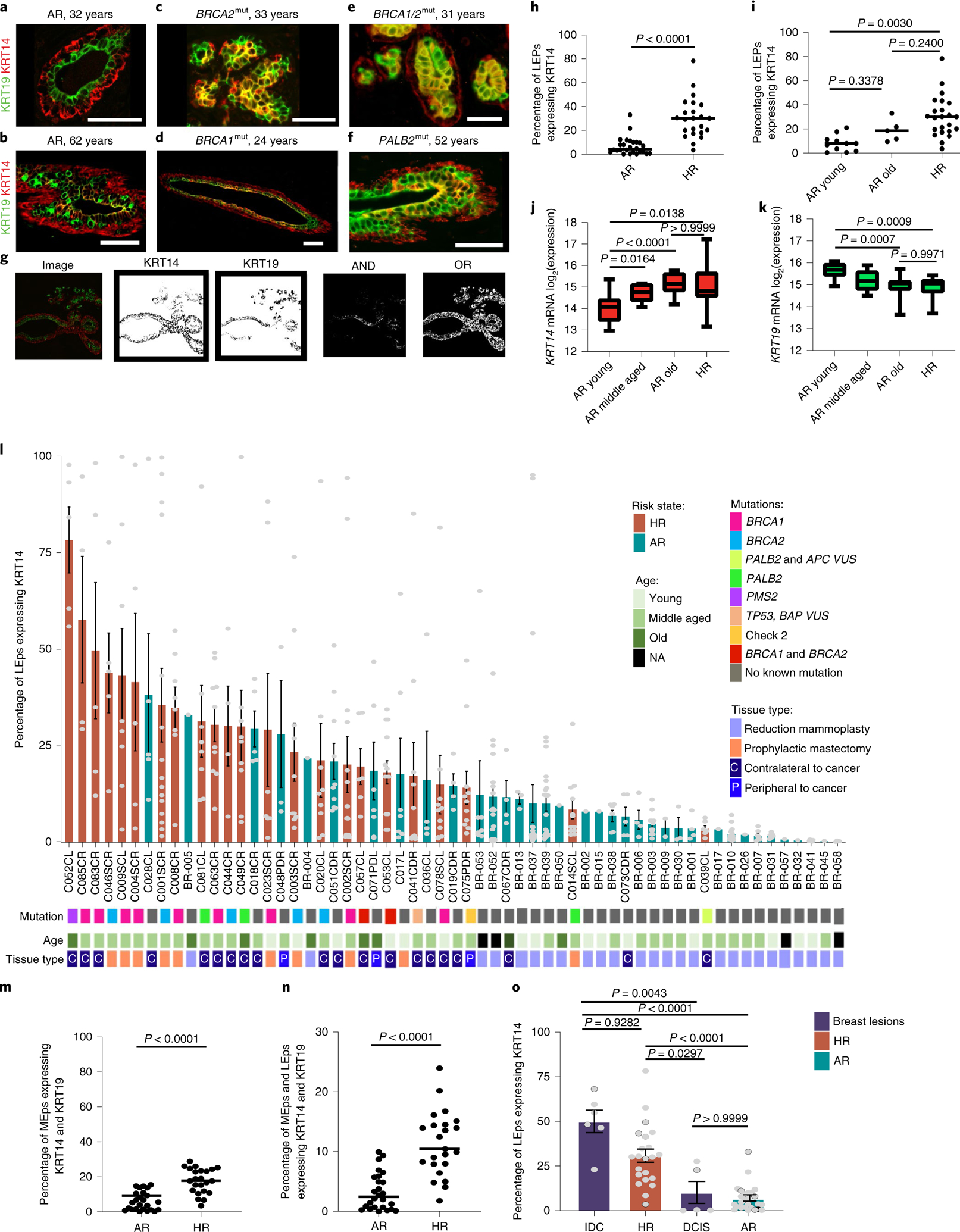
HR LEps coexpress KRT14 along with KRT19 resembling aged LEps. **a**–**f**, Immunofluorescent images of primary human mammary ducts stained for KRT19 (green) and KRT14 (red) from a 32-year-old AR woman (**a**), a 62-year-old AR woman (**b**), a 33-year-old woman harboring a *BRCA2* mutation (*BRCA2*^mut^) (**c**), a 24-year-old woman harboring a *BRCA1*^mut^ (**d**), a 31-year-old woman harboring *BRCA1* and *BRCA2* mutations (**e**) and a 52-year-old woman harboring a *PALB2*^mut^ (**f**). Scale bars, 50 μm. **g**, A schematic of the method used in quantification and analysis of immunofluorescent images. **h**, Dot plot of the percentage of KRT19^+^ LEps expressing KRT14 in HR (*n* = 23) and AR (*n* = 26) samples. The *P* value was computed using a two-sided Mann–Whitney *U*-test. **i**, Dot plot of the percentage of KRT19^+^ LEps expressing KRT14 in HR (*n* = 23), AR younger (≤35 years, *n* = 11) and AR older (>55 years, *n* = 5) strains. The *P* values were computed using one-way ANOVA adjusted for multiple comparisons using Tukey’s post-hoc test. **j**,**k**, The log_2_(expression) of *KRT14* (**j**) and *KRT19* (**k**) mRNA in passage 4 LEps. The outlines of the boxes represent the first and third quartiles. The vertical line inside the boxes represents the median, and the whiskers go from each quartile to the minimum and maximum values. The *P* values were computed using Welch’s ANOVA test adjusted for multiple comparisons with Dunnett’s T3 post-hoc test. **l**, Waterfall plot of the percentage of KRT19^+^ LEps that express KRT14 in HR and AR strains mapped for mutation, age and tissue type. Age groups are as follows: young, ≤35 years; middle aged, >35 years and ≤55 years; and old, >55 years. **m**, Dot plot of the percentage of KRT14^+^ MEps expressing KRT19 fluorescent signal in HR and AR samples. **n**, Dot plot of the percentage of KRT19^+^ LEps and KRT14^+^ MEps expressing KRT14 and KRT19 in HR and AR samples detected by immunofluorescence. In **m** and **n**, the *P* values were computed using a two-sided Mann–Whitney *U*-test. **o**, Waterfall plot of the percentage of KRT19^+^ LEps that express KRT14 in all HR strains, all AR strains and tissue sections of premalignant and malignant breast lesions. The *P* values were computed using the Kruskal–Wallis test adjusted for multiple comparisons using Dunn’s post-hoc test. In **l** and **o**, the edges of the bars represent the means and the error bars represent the s.e.m. The gray dots in **l** represent data from each image taken per strain. The gray dots in **o** represent the average of images taken per strain/sample in the groups listed. At least two sections from each individual were stained and analyzed independently with similar results. The number of different individuals representing each group are as follows: **a**, 11; **b**, five; **c**, five; **d**, nine; **e**, two; **f**, three.

Normal breast tissue was collected from HR and AR women undergoing breast reduction or prophylactic mastectomies (Supplementary Table 1). KRT14 and KRT19 protein expression in AR (*n* = 26, mean age = 38.5 years, median age = 39 years) and HR (*n* = 23, mean age = 45.5 years, median age = 48 years) tissue sections and RNA expression in epithelial cells were examined. In tissue sections from AR younger women, luminal epithelial cells (LEps) expressed KRT19 and myoepithelial cells (MEps) expressed KRT14 in a mutually exclusive relationship, although some heterogeneity existed at all ages (Fig. 1a). However, LEps in older AR women gained expression of KRT14, consistent with our previous findings^6,7^ (Fig. 1b). Representative images of tissue sections are shown from HR carriers of *BRCA2* (Fig. 1c), *BRCA1* (Fig. 1d), *BRCA1* and *BRCA2* (Fig. 1e), and *PALB2* mutations (Fig. 1f), all of which expressed KRT14 in the KRT19^+^ LEps. We developed an image analysis pipeline to quantify fluorescent signals corresponding to KRT14 and KRT19 expression in epithelial cells in immunofluorescent images of primary tissue sections (Fig. 1g). In HR epithelia (*n* = 23), 31% of KRT19^+^ LEps also expressed KRT14, compared with 7% of LEps in the AR (Mann–Whitney *U*-test, *P* < 0.0001; Fig. 1h). Younger (<35 years) AR (*n* = 11) epithelia had significantly fewer KRT14^+^ LEps than older AR (*n* = 5) or HR epithelia of any age, whereas there was no difference between older AR tissues and HR tissues of any age (Fig. 1i). Expression of *KRT14* messenger RNA was significantly higher in older AR LEps (*n* = 14) compared with younger (*n* = 17) and middle-aged (*n* = 7) AR LEps, but did not differ from HR samples (*n* = 13) (Fig. 1j). HR LEps had significantly more *KRT14* mRNA compared with younger AR LEps (Fig. 1j). Expression of *KRT19* mRNA in HR LEps (*n* = 13) did not differ from that of older AR LEps (*n* = 14) but was significantly less than that of younger and middle-aged AR LEps (*n* = 7) (Fig. 1k). Waterfall plots showed the overall trend of higher KRT14 expression in KRT19^+^ LEps in HR breast tissue mapped for each mutation, age group and tissue type (Fig. 1l). In our sample set, women with a germline *BRCA1*^mut^ tended to exhibit the highest KRT14 expression in LEps, relative to other mutation carriers (Fig. 1l). The increased proportion of KRT14^+^ LEps in HR tissue was independent of chronological age (Spearman’s *ρ* = 0.047, *P* = 0.83), whereas in AR tissue this increase was correlated with age (Spearman’s *ρ* = 0.37, *P* = 0.0334).

**Extended Data Fig. 1 |:**
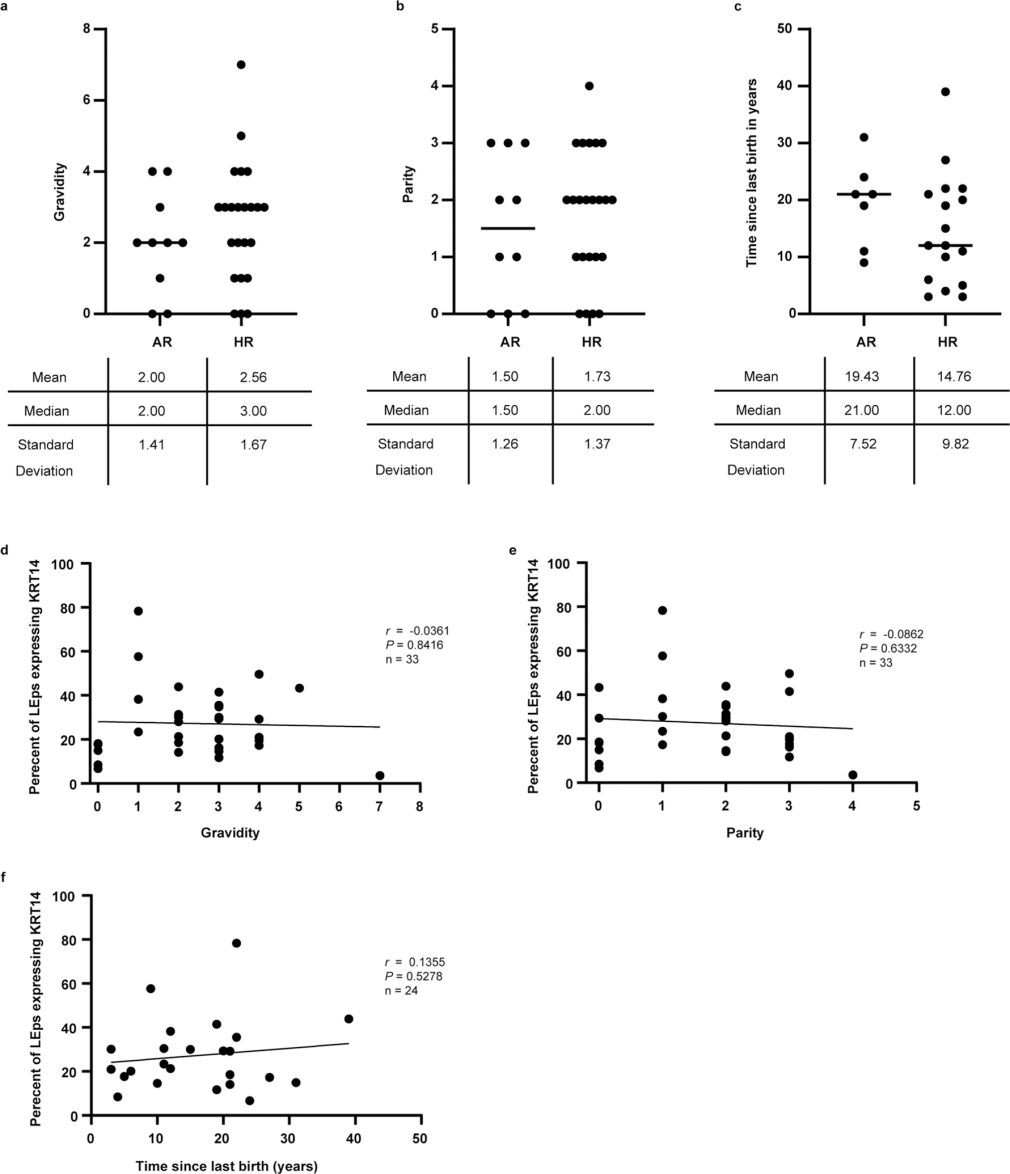
Parity status does not impact loss of lineage fidelity in LEps. (a-c) dot plots representing the distribution of (a) gravidity, (b) parity, and (c) time since last childbirth in years between average-risk (AR) and high-risk (HR) women included in this study. The lines on the plots represent the medians. (d-f) Correlation plots investigating the association of (d) gravidity, (e) parity, and (c) time since last childbirth in years and the percentage of luminal epithelial cells (LEps) expressing KRT14. The plots report Pearson correlation coefficient (r) and *P* values that were determined by a two tailed simple linear regression. The lines on the plots represent the best fit line computed via regression analysis.

As pregnancy affects breast cancer risk differentially, depending on the age of the individual at their first pregnancy and the time of their last childbirth^19–21^, we assessed parity and gravidity statuses along with the time since last childbirth (when available) in relation to KRT14 changes in LEps. Loss of lineage fidelity in LEps was not associated with childbirth status because the mean values of parity, gravidity and time since last birth did not differ between risk groups and did not correlate with increased KRT14 expression in LEps (Extended Data Fig. 1).

To assess loss of lineage fidelity in MEps, we examined KRT14 and KRT19 expression in MEps. Of KRT14^+^ MEps within HR epithelia 18% (*n* = 23) also expressed KRT19, suggesting loss of lineage fidelity in MEps (Fig. 1m). Loss of lineage fidelity seemed to be a characteristic feature of HR epithelia; among all epithelial cells (LEps and MEps), 11% of cells in HR epithelia expressed staining for both keratins, compared with 3% of cells in AR epithelia (*n* = 26) (Mann–Whitney *U*-test, *P* < 0.0001; Fig. 1n).

We next examined the frequency of KRT14 expression in KRT19^+^ cells in premalignant and malignant breast lesions, such as ductal carcinoma in situ (DCIS; *n* = 5) and invasive ductal carcinomas (IDC; *n* = 6). The ages for the women who had DCIS ranged from 21 years to 54 years (median = 37 years) and for those who had IDC ranged from 34 years to 51 years (median = 47 years). In IDC, almost 50% of KRT19^+^ cells also expressed KRT14, compared with 31% of KRT19^+^ LEps in HR epithelia and 7% of KRT19^+^ LEps in AR epithelia (Kruskal–Wallis test, *P* < 0.0001; Fig. 1o). Proportions of KRT19^+^ cells expressing KRT14 in DCIS lesions trended higher than those from AR tissues, but the result was not significant (Fig. 1o). In summary, HR breast epithelia of women harboring cancer-predisposing mutations had significantly higher levels of KRT14 expression in their LEps compared with AR, a property of LEps observed in both women of advanced age and women with breast cancer.

### An abundance of luminal cells in HR epithelia.

**Extended Data Fig. 2 |:**
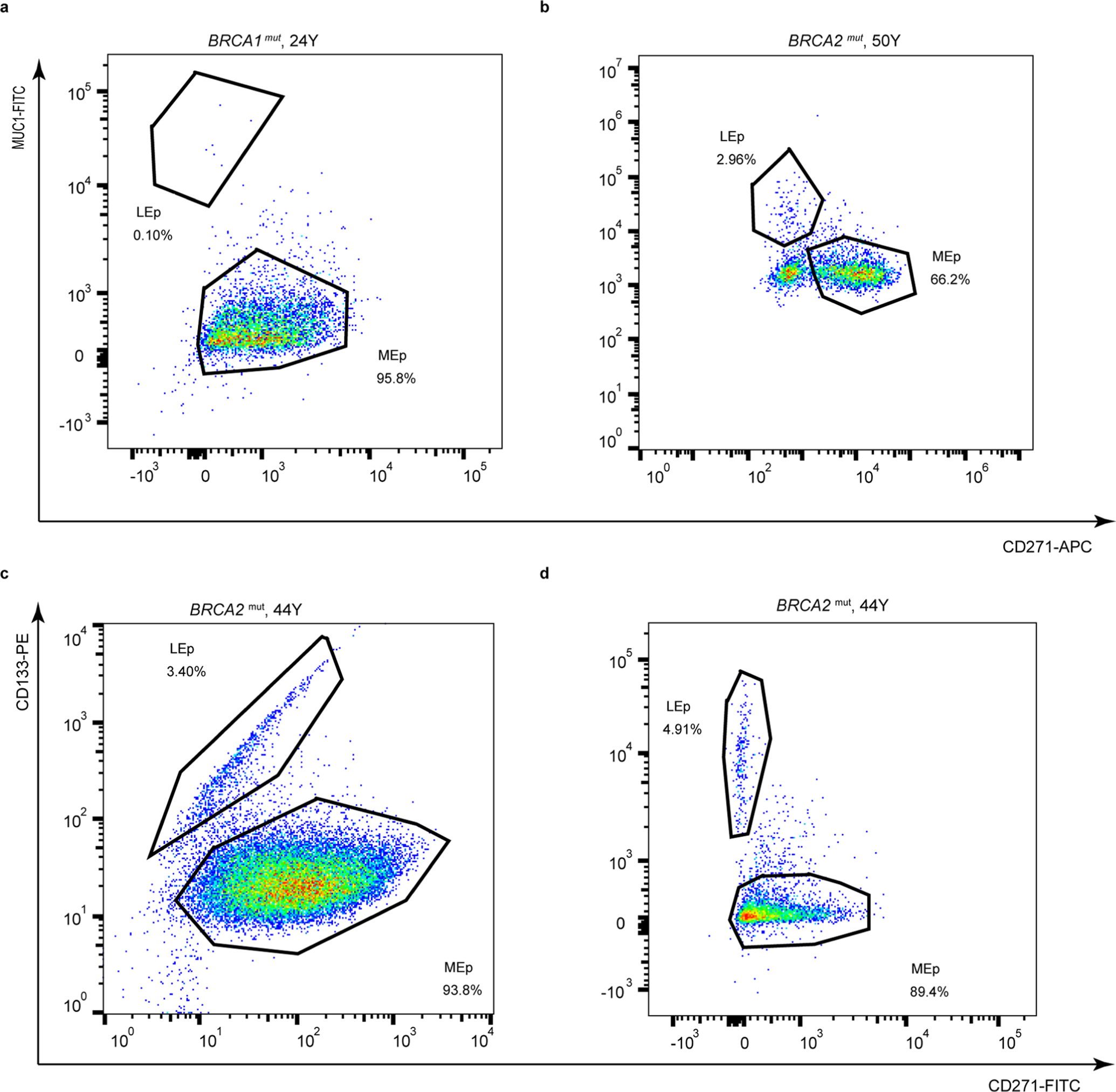
High-risk epithelial strains with low LEp populations. Flow cytometry analysis of passage four epithelia stained for (a-b) CD271(MEp marker) and MUC1(LEp marker) or (c-d) CD271 and CD133 (LEp marker) from HR women carrying (a) *BRCA1* or (b-d) *BRCA2* mutations.

**Fig. 2 |:**
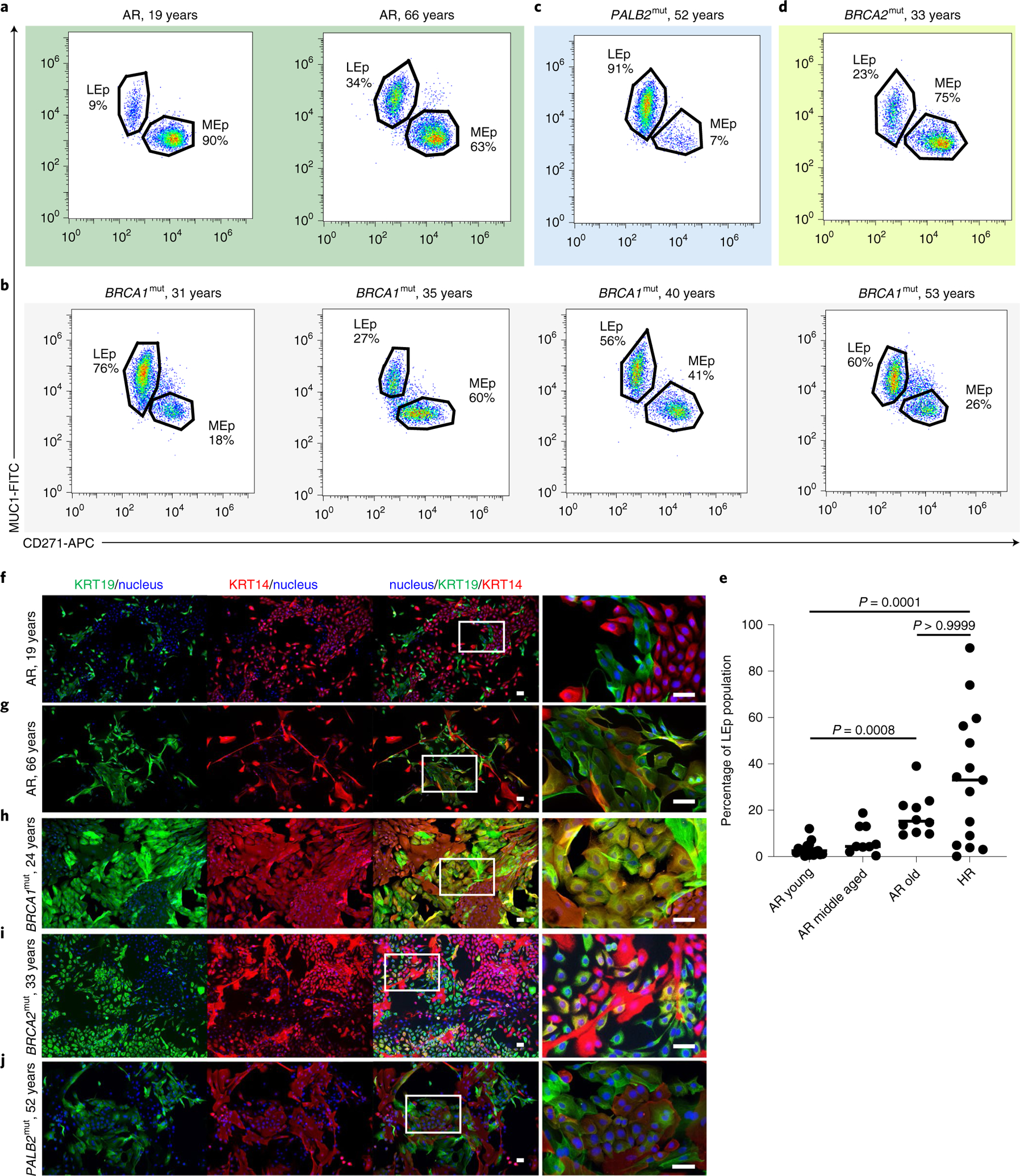
HR cultured mammary epithelial cells show luminal expansion with a basal phenotype. **a**–**d**, Flow cytometry analysis of passage 4 epithelia stained for CD271 and MUC1 from an AR 19-year-old woman and an AR 66-year-old woman (**a**), HR women of different ages (31, 35, 40 and 53 years) harboring *BRCA1* mutations (**b**), an HR 52-year-old woman harboring a *PALB2* mutation (*PALB2*^mut^) (**c**) and an HR 33-year-old woman harboring a *BRCA2*^mut^ (**d**). **e**, Dot plot of the percentage of LEp populations from passage 4 epithelial strains derived from AR younger women (≤35 years, *n* = 17), AR middle-aged women (36–55 years, *n* = 9), AR older women (>55 years, *n* = 10) and HR women (*n* = 15). The median of each sample is indicated by a horizontal line. The *P* values were computed using the Kruskal–Wallis test adjusted for multiple comparisons with Dunn’s post-hoc test. **f**–**j**, Immunofluorescence staining of passage 4 cultured epithelial cells stained for KRT19 (green), KRT14 (red) and Hoechst (blue) from an AR 19-year-old woman (**f**), an AR 66-year-old woman (**g**), an HR 24-year-old woman harboring a *BRCA1*^mut^ (**h**), an HR 33-year-old woman harboring a *BRCA2*^mut^ (**i**) and a 52-year-old woman harboring a *PALB2*^mut^ (**j**). The image on the right-hand side of each panel is a magnification of the area outlined by the white rectangle in the merged image. Scale bars, 50 μm. Experiments in **f**–**j** were repeated three times independently with similar results.

We next determined whether proportions of LEps and MEps differed between HR and AR human mammary epithelial cells (HMECs). Flow cytometry was used to assess the relative expression of the MEp marker CD271 (neural growth factor receptor) and LEp marker CD227 (sialomucin 1 (MUC1)) on HMECs from age-matched AR (*n* = 17 younger, 9 middle aged and 10 older) and HR (*n* = 15) women. AR younger strains varied from 0.2% to 12% LEps (median = 2.5%) and increased to 9–39% in older strains (median = 15.3%; Kruskal–Wallis test, *P* = 0.0008), consistent with our previous findings^6,7^ (Fig. 2a,e). We observed heterogeneity among HR strains: 4 of 15 strains had less than 5% LEps (Extended Data Fig. 2), but 11 of 15 (73%) HR strains had significantly larger proportions of LEps than what we would have predicted based on age alone (range = 0.1–90%, median = 33%; Fig. 2b,e). The most profound example of LEp expansion was in a germline *PALB2*^mut^ carrier that reached up to 91% LEps (Fig. 2c). HMECs harboring a germline *BRCA1*^mut^ had LEp populations ranging from 1% to 60% (median = 41.7%) (Fig. 2b); in HMECs with a germline *BRCA2*^mut^ LEps ranged from 3% to 28% (median = 4.9%) (Fig. 2d and Extended Data Fig. 2). Cultured HMECs were examined for KRT14 and KRT19 expression by immunofluorescence. LEps and MEps in younger AR HMECs expressed KRT19 and KRT14 in a mutually exclusive manner (see for example, Fig. 2f), whereas, in HR and older AR HMECs, KRT19^+^ LEps also expressed KRT14 (see for example, Fig. 2g–j). HR HMECs exhibited expanded proportions of LEps with increased KRT14 expression even at younger ages.

### HR progenitors have differentiation defects and a basal bias.

**Extended Data Fig. 3 |:**
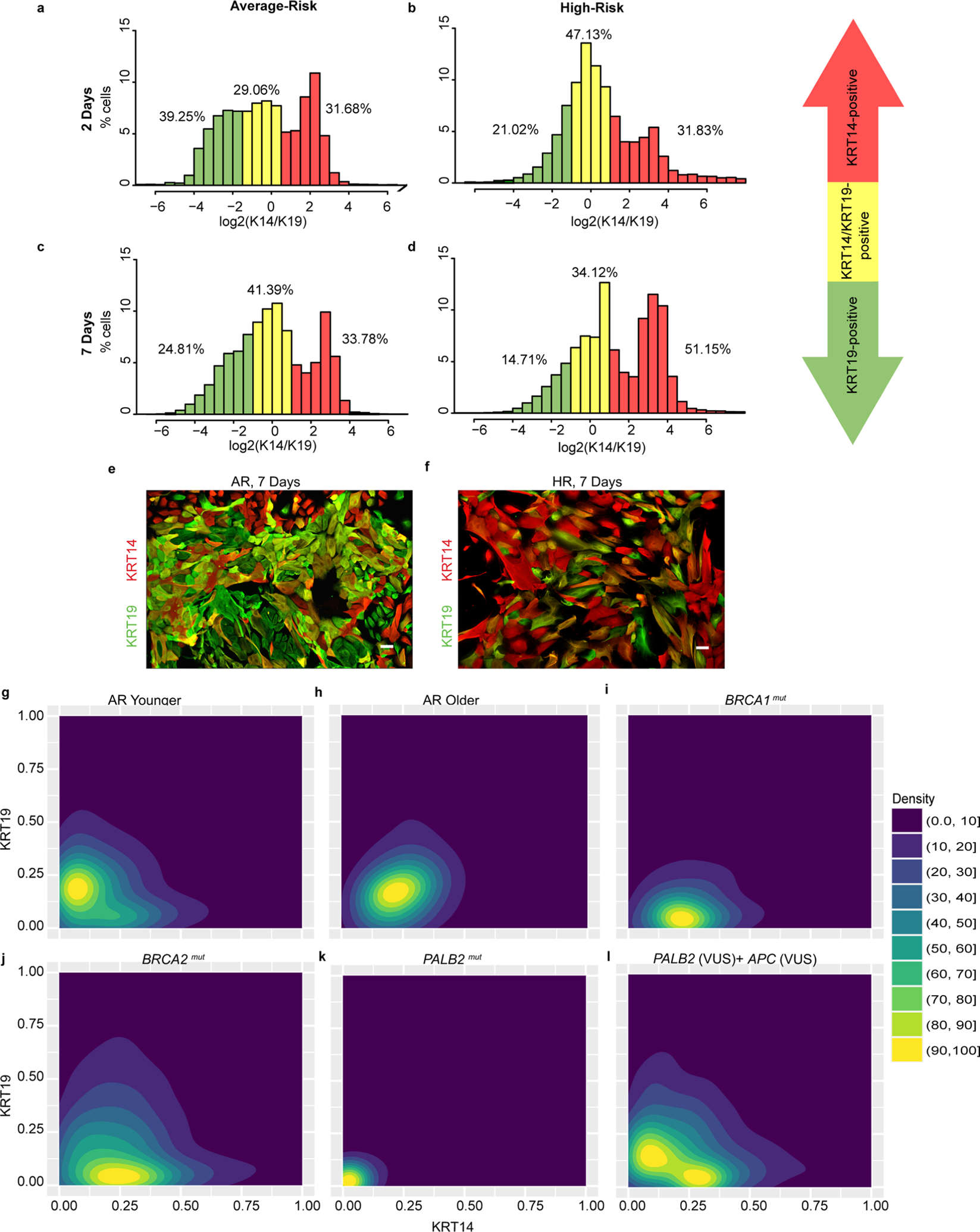
High-risk mammary epithelial progenitors with various mutations show basal differentiation biases after 7 days of culture. (a-d) Histograms of cKit-enriched cells stained with KRT14 and KRT19 that were fixed after 2 days of culture from (a) AR women with no predisposing mutations and (b) HR women, and after 7 days of culture from (c) AR women and (d) HR women. (e-f) Immunofluorescent images of cKit-enriched cells stained for KRT19 (green) and KRT14 (red) that were fixed after 7 days of culture from (e) an AR woman (40 y) and (f) an HR woman harboring a germline *BRCA2* mutation (44 y). (g-l) Density contour plots of KRT14 and KRT19 mean fluorescent intensity signals in cKit-enriched cells that were fixed after 7 days of culture from (g) AR younger women, (h) AR older women, and HR women harboring germline mutations of (i) *BRCA1*, (j) *BRCA2*, (k) *PALB2* and (l) *PALB2* (VUS)+*APC* (VUS). Scale bars = 50 μm. Abbreviations are as follows: VUS, variant of unknown significance. Experiments in e-f were repeated three times independently with similar results. The number of cell strains used in each experiment representing the two groups are as follows: e, five; f, thirteen.

**Extended Data Fig. 4 |:**
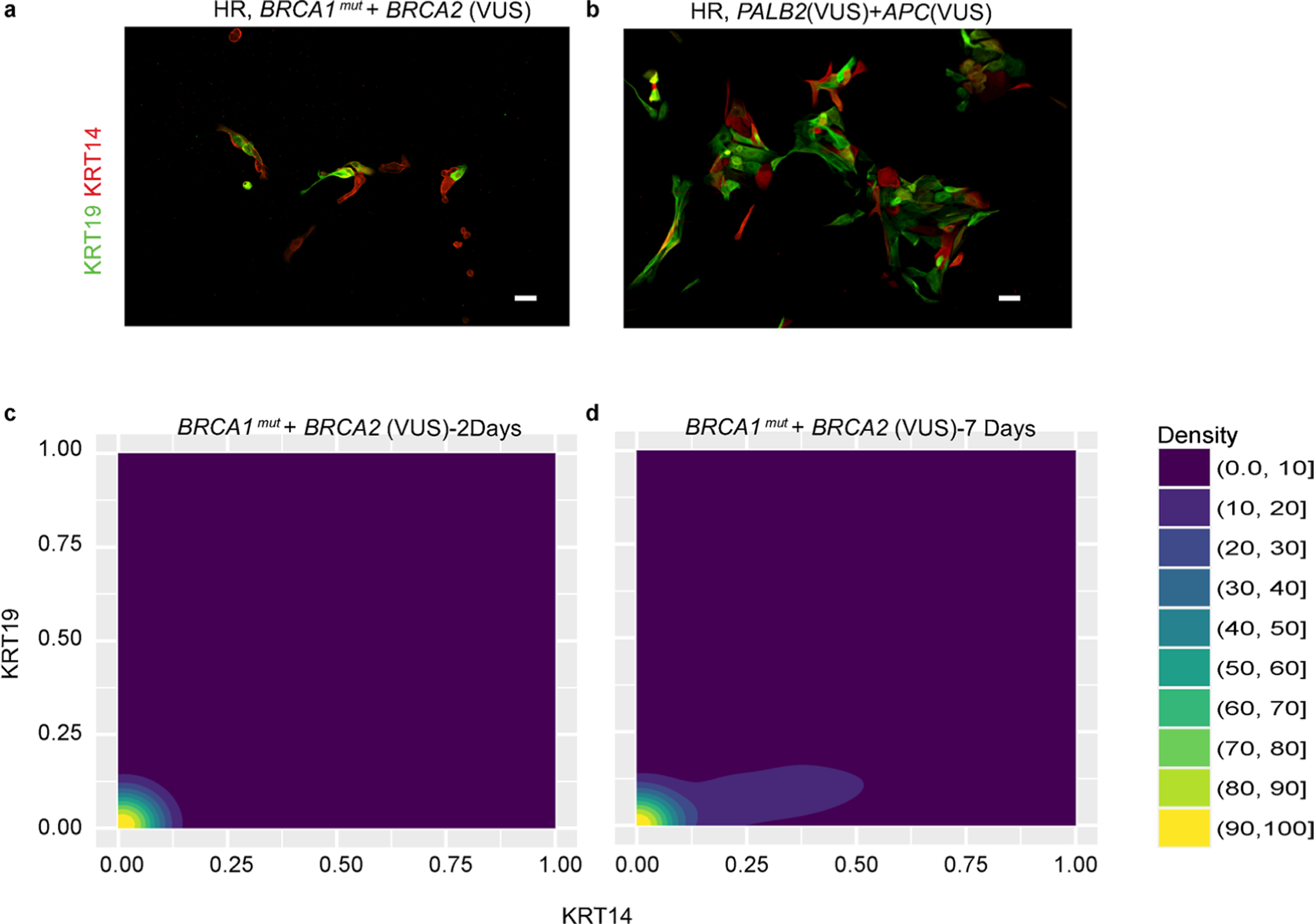
Differentiation patterns from high-risk mammary epithelial progenitors that had a VuS. (a-b) Immunofluorescent images of cKit-enriched cells stained for KRT19 (green) and KRT14 (red) that were fixed after 2 days of culture from (a) an HR woman harboring a germline *BRCA1* mutation with a *BRCA2* VUS and (b) an HR woman harboring a *PALB2*(VUS) and *APC*(VUS). (c-d) Density contour plots of KRT14 and KRT19 mean fluorescent intensity signals in cKit-enriched cells from an HR woman harboring germline mutations of (b) *BRCA1*+*BRCA2*(VUS) that were either fixed after (c) 2 days or (d) 7 days of culture. Experiments in a-b were repeated twice with similar results. Scale bars = 50 μm.

**Fig. 3 |:**
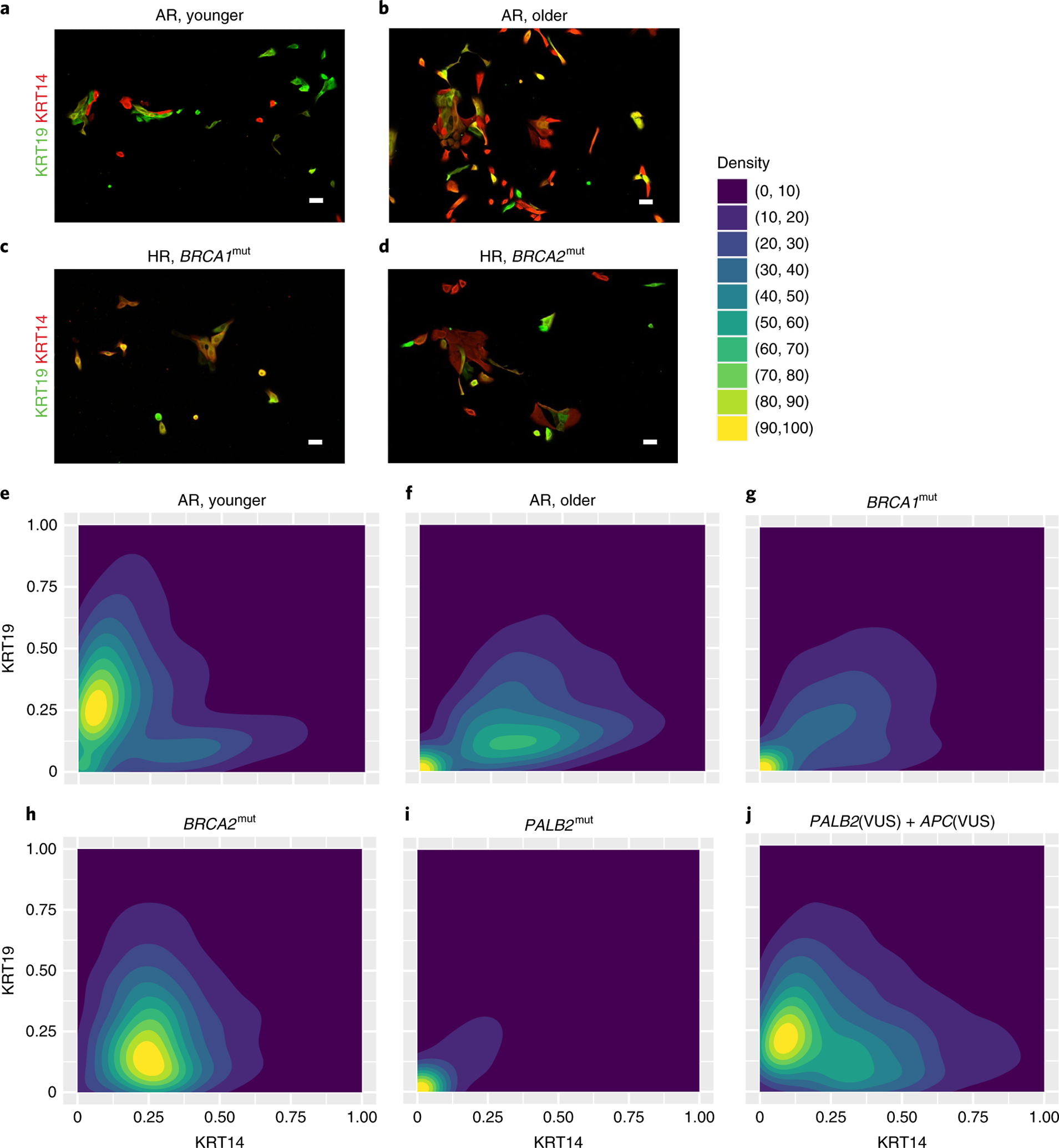
HR mammary epithelial progenitors show a differentiation defect and a basal differentiation bias. **a**–**d**, Immunofluorescent images of cKit-enriched cells stained for KRT19 (green) and KRT14 (red) that were fixed at 2 d of culture from an AR younger woman (**a**), an AR older woman (**b**), an HR 35-year-old woman harboring a *BRCA1* mutation (*BRCA1*^mut^) (**c**) and an HR 44 year-old woman harboring a *BRCA2*^mut^ (**d**). Scale bars, 50 μm. **e**–**j**, Density contour plots of KRT14 and KRT19 mean florescent intensity signals in cKit-enriched cells that were fixed after 2 d of culture from AR younger women (**e**), AR older women (**f**) and HR women harboring germline mutations of *BRCA1* (**g**), *BRCA2* (**h**), *PALB2* (**i**) and *PALB2*(VUS) + *APC*(VUS) (**j**). Experiments in **a**–**d** were repeated three times independently with similar results. The number of individuals with cKit^+^ were derived from representing each group as follows: **a**, three; **b**, two; **c**, eight; **d**, six.

To investigate whether epithelial progenitors from HR epithelia exhibited differentiation biases, we performed clonal differentiation assays on receptor tyrosine kinase cKit (CD117)-expressing cells enriched by FACS. Mammary epithelial cells enriched for high expression of cKit are capable of multilineage differentiation, but there is a luminal bias^7,22^. After FACS enrichment, cKit^+^ cells were attached to cover slips at clonal density, and daughter cells in the resulting colonies were assayed by immunofluorescence for the differentiation markers KRT14 and KRT19 after either 2 d or 7 d. Marker-based watershed segmentation identified single cells in images, followed by measurement of the mean fluorescent signal that corresponded to KRT14 or KRT19 expression. The undifferentiated state of cKit progenitors is depicted by the dual expression of low levels of both lineage markers, usually resulting in cells appearing in the lower left corner of dot or contour plots that show KRT intensities. The cKit-enriched cells from AR (*n* = 5) and HR (*n* = 18: eight *BRCA1*^mut^, six *BRCA2*^mut^ and four *PALB2*^mut^) cells were examined (Fig. 3a–d and Extended Data Fig. 3a–f). The mean age of the strains harboring a *BRCA1*^mut^ was 39.6 years (median = 36 years), the mean age of the strains harboring a *BRCA2*^mut^ was 52 years (median = 52 years) and the mean age of the strains harboring a *PALB2*^mut^ was 54.5 years (median = 54.5 years). Younger AR cKit^+^ cells gave rise to distinct populations of KRT19^+^ or KRT14^+^ cells after 2 d (Fig. 3a,e and Extended Data Fig. 3a). Older AR cells had a basal differentiation bias; most progeny expressed some KRT14; the age-dependent differentiation distributions in AR strains replicated our previous findings^7^ (Figs. 3b,f). The distribution of progeny arising from HR cKit^+^ progenitors after 2 d differed significantly from that of AR cKit^+^ progenitors (χ^2^ test, *P* < 0.0001); almost 50% of HR cKit^+^ cells remained undifferentiated compared with 29% of AR cKit^+^ cells (Extended Data Fig. 3a,b). This undifferentiated state was the prominent feature of cKit^+^ cells with *BRCA1* mutations (Fig. 3c,g and Extended Data Fig. 4a,c). Progeny of cKit^+^ cells with *BRCA2* mutations differentiated with a basal bias, that is, they all expressed some KRT14, but there was discernably more KRT19 expression compared with *BRCA1*^mut^ carriers (Fig. 3d,h). Qualitatively, the *BRCA2*^mut^ phenotype bears more resemblance to AR older cKit^+^ cells. The cKit^+^ cells harboring a known pathogenic *PALB2* variant failed to differentiate (Fig. 3i), but the carrier of a *PALB2* variant of unknown significance (VUS) had a differentiation pattern similar to that of AR younger cells, with progeny showing mutually exclusive populations of KRT19^+^ and KRT14^+^ cells (Fig. 3j and Extended Data Fig. 4b).

After 7 d, HR cKit^+^ cells had given rise to progeny that were 51% KRT14^+^ compared with 34% of AR cKit^+^ cell progeny (χ^2^ test, *P* < 0.0001) (Extended Data Fig. 3c–f,i–l). Differentiation patterns of HR cKit^+^ cells after 7 d, categorized by mutation, are shown in Extended Data Figs. 3i–k and 4d. The cKit-enriched cells with *BRCA1* and *BRCA2* mutations differentiated into mostly KRT14^+^ cells. The cKit-enriched cells with *PALB2* mutations failed to differentiate even after 7 d (Extended Data Fig. 3k). However, cKit^+^ cells harboring a *PALB2*(VUS) had a distribution closer to what is characteristic of AR younger cKit^+^ cells (Extended Data Fig. 3l).

Progeny of cKit^+^ progenitor cells from HR epithelia exhibited a pattern of basal differentiation bias that seemed to be independent of the specific germline mutation. However, *BRCA2*^mut^ progenitors eventually gave rise to KRT14^+^ progeny that also expressed KRT19, whereas *PALB2*^mut^ carriers showed a striking delay in differentiation; most of the cells resided as keratin double-positive cells for a prolonged period in this assay.

### High risk epithelia map to aging, inflammatory and cancer genes.

**Fig. 4 |:**
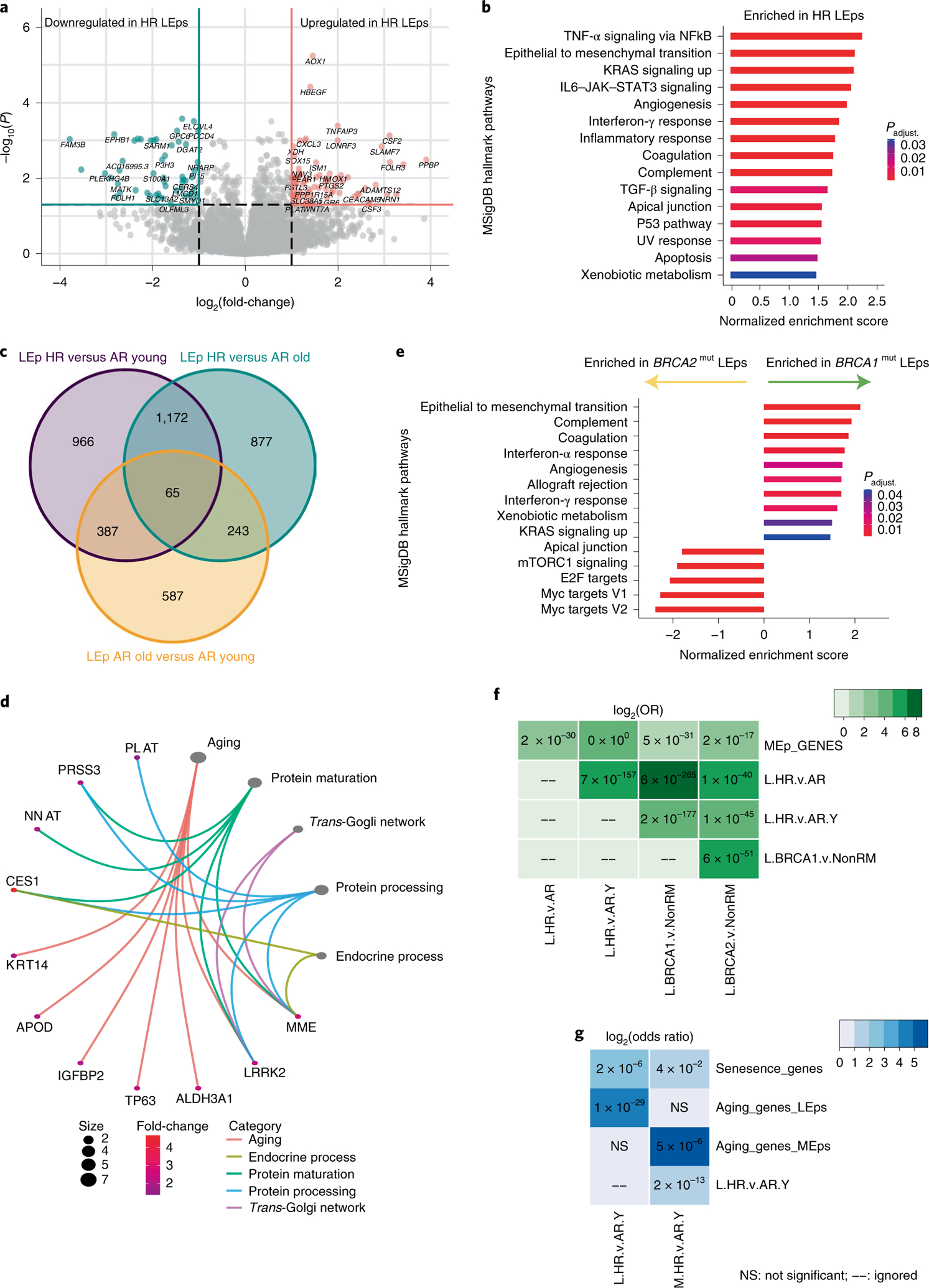
Transcriptomes of HR LEps resemble aged LEps and map to inflammatory and cancer-related pathways. **a**, Volcano plot of DE genes in HR compared with AR LEps. **b**, MSigDB hallmark gene sets enriched in HR LEps compared with AR LEps. TGF-β, transforming growth factor β. **c**, Venn diagram of DE genes of three comparisons: HR versus AR younger (≤35 years) LEps, HR versus AR older (>55 years) LEps and AR older versus AR younger LEps. **d**, GO terms overrepresented in common genes upregulated in both HR and AR older LEps. The size of the circles reflects the number of genes per term. *P*_adjust_. is the adjusted *P* value for multiple comparisons. **e**, MSigDB hallmark gene sets enriched in LEps harboring *BRCA1* versus *BRCA2* mutations. **f**,**g**, Matrices of genes overlapping between several comparisons. Numbers in the matrix represent *P* values; the color gradient is the log_2_(OR) of the overlap. The *P* values were computed using Fischer’s exact test within the GeneOverlap package for each pair of gene lists compared in a one-sided manner (alternative = greater). L.HR.v.AR, genes upregulated in HR LEps compared with AR LEps; L.HR.v.AR.Y, genes upregulated in HR LEps compared with AR younger LEps (≤35 years); L.BRCA1.v.Non.RM, genes upregulated in LEps harboring *BRCA1* mutations compared with AR LEps that do not harbor any mutations collected from reduction mammoplasties; L.BRCA2.v.Non.RM, genes upregulated in LEps harboring *BRCA2* mutations compared with AR LEps that do not harbor any mutations collected from reduction mammoplasties; M.HR.v.AR.Y, genes upregulated in HR MEps compared with AR younger MEps (≤35 years).

We next compared transcriptomes of LEps and MEps isolated from HR women (*n* = 11; six *BRCA1*^mut^, three *BRCA2*^mut^ and two *PALB2*^mut^) and age-matched LEps and MEps isolated from AR women (*n* = 9). In total, 336 genes were differentially expressed (DE) between HR LEps and AR LEps (212 upregulated and 124 downregulated; adjusted *P*-value cutoff <0.05; Fig. 4a and Supplementary Table 2). Using the Molecular Signatures Database (MSigDB), DE genes in HR LEps were enriched for hallmark gene sets of EMT and inflammation, including gene sets for tumor necrosis factor-α (TNF-α) via nuclear factor κ-light-chain-enhancer of activated B cells (NF-κB), interleukiin (IL)-6–JAK–STAT3 and interferon-γ signaling (false discovery rate (FDR) <0.05; Fig. 4b). We next evaluated whether the age-dependent gene expression changes in AR epithelia were also a feature of HR epithelia. We merged the above HR transcriptome dataset with data derived from AR LEps and MEps isolated from younger (*n* = 16) and older (*n* = 14) reduction mammoplasties; 452 genes were common in the DE genes between HR and AR younger LEps and between AR older and AR younger LEps in the same direction, suggesting that these are common risk genes, whether due to aging or underlying genetic risk (Fig. 4c and Supplementary Table 3). Gene ontology (GO) terms overrepresented by these common risk genes included aging and protein-processing terms (Fig. 4d). To investigate potential differences within the HR group, we performed DE analysis between LEps from *BRCA1*^mut^ and *BRCA2*^mut^ carriers. *BRCA1*^mut^ LEps were enriched for MSigDB hallmark gene sets of EMT, complement, coagulation, angiogenesis, and interferon-α and -γ responses, whereas *BRCA2*^mut^ LEps were enriched for hallmark gene sets of MYC targets, versions 1 and 2, E2F targets and mammalian target of rapamycin complex 1 (mTORC1) signaling (Fig. 4e). These data provide evidence that there are common sets of genes expressed in LEps from both AR older women and HR women who carry a germline mutation that increases their risk for breast cancer at any age.

We next examined whether age-dependent and lineage-specific gene signatures overlapped with gene sets enriched in HR epithelial cells. Gene overlap was assessed by odds ratio (OR) analysis, whereby values <1 indicate no overlap between two gene sets and values >1 indicate a strong overlap^23^. MEp-specific genes (Supplementary Table 4) overlapped with genes upregulated in HR LEps compared with age-matched AR LEps with an OR of 7.7 (*P* < 0.0001), and with genes upregulated in HR LEps compared with AR younger LEps with an OR of 27.3 (*P* < 0.0001) (Fig. 4f and Supplementary Table 5). This was echoed by genes upregulated in *BRCA1*^mut^ LEps and *BRCA2*^mut^ LEps compared with AR LEps from reduction mammoplasties (Fig. 4f). This indicates that HR LEps express genes of the MEp/basal lineage, suggesting loss of lineage fidelity. Genes upregulated in HR LEps overlapped with MSigDB senescence signature genes with an OR of 4.1 (*P* = 0.016; Fig. 4g). In addition, we established an aging signature in LEps by selecting the genes upregulated in older LEps compared with younger LEps (Benjamini–Hochberg-adjusted *P* < 0.05; Supplementary Table 6). We similarly established an aging signature in MEps (Supplementary Table 7). Genes upregulated in HR LEps overlapped with the aging LEp gene signature with an OR of 20 (*P* < 0.0001; Fig. 4g and Supplementary Table 8). Together this indicated that HR LEps possess transcriptomic features of accelerated aging and enrichment of gene pathways known to be involved in breast cancer and inflammation.

**Fig. 5 |:**
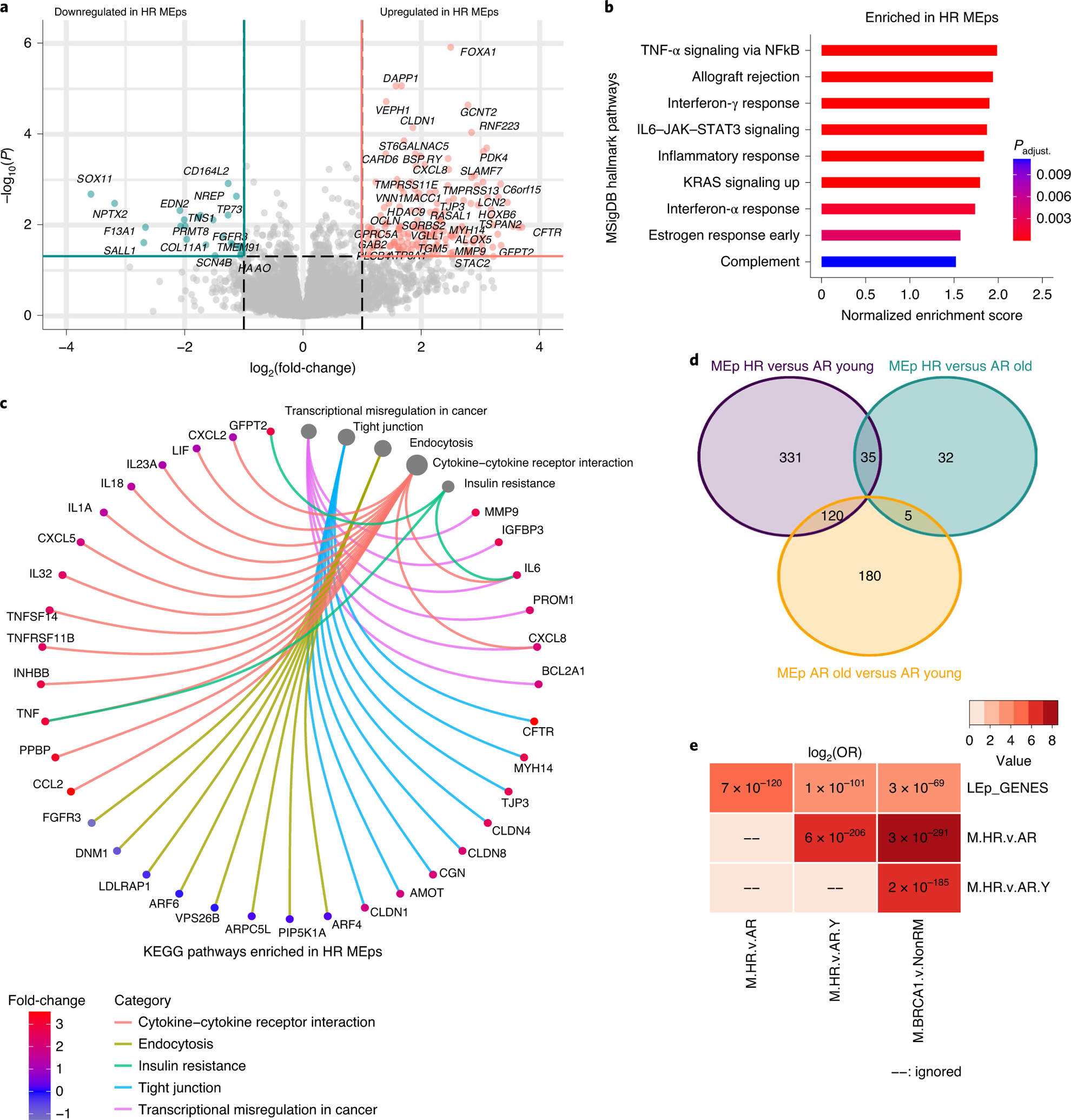
Transcriptomes of HR MEps resemble aged MEps and map to inflammatory and cancer-related pathways. **a**, Volcano plot of DE genes in HR compared with AR MEps. **b**, MSigDB hallmark gene sets enriched in HR MEps compared with AR MEps. **c**, KEGG pathways overrepresented in HR MEps compared with AR MEps. The size of the circles reflects the number of genes per pathway. **d**, Venn diagram of DE genes of three comparisons: HR versus AR younger (≤35 years) MEps, HR versus AR older (>55 years) MEps and AR older versus AR younger MEps. **e**, A matrix of genes overlapping across several comparisons. Numbers in the matrix represent *P* values whereas the color gradient is the log_2_(OR) of the overlap. The *P* values were computed using Fischer’s exact test within the GeneOverlap package for each pair of gene lists compared in a one-sided manner (alternative = greater). M.HR.v.AR, genes upregulated in HR MEps compared with AR MEps; M.HR.v.AR.Y, genes upregulated in HR MEps compared with AR younger MEps (≤35 years); M.BRCA1.v.Non.RM, genes upregulated in MEps harboring *BRCA1* mutations compared with AR MEps that do not harbor any mutations collected from reduction mammoplasties.

In MEps, 346 genes were DE in HR cells compared with AR cells (250 upregulated and 96 downregulated; Fig. 5a and Supplementary Table 9). HR MEps were enriched for MSigDB hallmark gene sets that are inflammatory (for example, TNF-α signaling and interferon-γ response) and promote aggressive biology (for example, KRAS signaling; Fig. 5b). KEGG pathways overrepresented in HR MEps included pathways affecting extracellular communication, such as tight junctions and cytokines, in addition to insulin resistance (Fig. 5c). Only 72 genes were DE between HR MEps and AR older MEps (Fig. 5d and Supplementary Table 10), which indicated similarity in their transcriptomes. Genes upregulated in HR MEps compared with both age-matched AR MEps and AR younger MEps overlapped with LEp-specific genes (Supplementary Table 11) with ORs of 25 and 12.3, respectively (*P* < 0.0001 for both; Fig. 5e and Supplementary Table 12). This suggested that HR MEps are enriched for some LEp-specific genes in their transcriptomes, consistent with a loss of lineage fidelity. Genes upregulated in HR MEps also overlapped with MSigDB senescence signature genes with an OR of 3.3 (*P* = 0.063; Fig. 4g and Supplementary Table 8). The aging signature in MEps that we established overlapped with genes upregulated in HR MEps with an OR of 60 (*P* < 0.0001; Fig. 4g and Supplementary Table 8). In summary, transcriptomes of HR epithelia were enriched for inflammatory and cancer-promoting pathways and overlapped with senescence and aging signature genes. HR LEps and MEps showed some enrichment for gene sets that are characteristic of the opposite epithelial lineage, which suggested that loss of lineage fidelity is a property shared by both HR epithelia and aged epithelia.

## Discussion

In the present study, we reported compositional, transcriptional and functional features consistent with accelerated biological aging in pathologically normal mammary epithelia that carried deleterious germline mutations, which are known to make women susceptible to breast cancer. Features of accelerated aging included loss of epithelial lineage fidelity, increased proportions of LEps that express proteins and genes typically associated with MEps, and a basal differentiation bias in cKit^+^ progenitors. It is speculated that the increased incidence of breast cancer in women who carry *BRCA1*, *BRCA2* or *PALB2* germline mutations is due to deficiencies in DNA damage repair^24,25^. Deficiencies in repairing various DNA insults could hasten accumulation of DNA aberrations compared with cells from AR individuals, driving a faster rate of biological aging compared with chronological age^3^. Therefore, it is reasonable to hypothesize that features of accelerated aging are a characteristic of mammary tissues that harbor such germline mutations. Indeed, women carrying germline *BRCA1* mutations exhibit features of accelerated aging in their ovaries, such as primordial follicle loss and diminished ovarian reserve^25–27^, and were reported to have earlier menopause than AR women^23^. Our HR HMEC strains and breast tissue sections were from both pre- and postmenopausal women, suggesting that these accelerated aging phenotypes are not necessarily dependent on hormone withdrawal. We propose that loss of lineage fidelity and acquisition of basal characteristics in LEps may be a hallmark emergent property of breast tissue that is more susceptible to cancer initiation, regardless of the specific source of underlying risk.

The acquired basal properties in LEps from pathologically normal mammary tissue with *BRCA1*, *BRCA2* or *PALB2* germline mutations manifested as increases in MEp-related intermediate filament protein and increased MEp, aging, EMT and senescence gene signatures. Other basal properties that were previously associated with *BRCA1*^mut^ epithelia include increased representation of vimentin and integrin-α_6_-expressing epithelia and KRT14-expressing LEps of acini derived in three-dimensional culture^28^. MEps are thought to be tumor suppressive through both production of basement membrane components and formation of a dynamic barrier that prevents invasion of cancer cells from the ducts out into the stroma^29,30^. Crucially, the HR and aged LEps do not appear to transdifferentiate into MEps, but they may gain access to some MEp functions. Perhaps LEps’ access to molecular programs that enable MEps to conduct their business in an extracellular matrix-rich microenvironment, and to move in and out of the stroma, is deleterious in the context of aging and HR states. For instance, compared with younger AR HMECs, HR and older HMECs showed an abundance of LEps when cultured on plastic. MEps proliferate faster than LEps on rigid surfaces such as tissue culture plastic^31^, so the success of HR and older LEps in culture suggests that they have acquired access to key gene programs required for success in a microenvironment that would normally be more ideal for basal cell types. Increased KRT14 expression in HR LEps may indicate access to molecular programs underlying aggressive biology. KRT14-expressing breast cancer cells were shown to lead the collective invasion process and to activate a basal gene program, which included *TP63* and *KRT5*, needed for the initial phases of metastasis in breast cancer cells^32^. Loss of lineage specificity was tracked in eight stages of lung cancer progression, leading to a subgroup of cells with high plasticity that were proliferative and drug resistant^33^. We speculate that the loss of the lineage fidelity in HR and aged epithelia is a precursor to losing lineage specificity, thought to be a key event in initiating cancer^34^.

Most HR cKit^+^ progenitors harboring *BRCA1* mutations resided as undifferentiated progenitor cells for a protracted period compared with AR controls in our experiments. When these progenitors differentiated eventually, there was a basal bias. The bias in HR cKit^+^ cells aligns conceptually with reports describing basal cancer cells of origin as luminal progenitor cells^9–12^, which were shown to be expanded in *BRCA1*^mut^ carriers^9^. Single-cell RNA-sequencing (RNA-seq) also revealed the expansion of cKit luminal progenitors with acquired basal properties in the tumors and adjacent tissue of *BRCA1*^mut^ carriers^12^. The differentiation defect we observed in HR cKit^+^ cells also aligns with the aberrant phenotype reported in luminal progenitors carrying a *BRCA1*^mut^ described previously^9^. Whereas we measured lineage biases based on markers of differentiation, Lim et al. characterized the aberrant phenotype by the ability of *BRCA1*^mut^ progenitors to form colonies independent of B27 supplement, which contained progesterone that was required for the growth of the normal progenitors^9^. In addition to the *BRCA1*^mut^ carriers, we showed that the basal differentiation bias was a feature of *BRCA2*^mut^ and also of older AR cKit^+^ progenitors. *PALB2*^mut^ samples failed to differentiate even after 7 d. The M87A medium used here does not contain progesterone and supports robust growth of AR and HR HMECs alike. M87A is supportive of multilineage epithelial growth, whereas we showed previously that defined serum-free medium, such as that used by Romijn et al.^35^, drives normal LEp into early stress-associated senescence^36^. Differentiation defects in cKit^+^ progenitors seem to be a common property of epithelia from women who are susceptible to cancer initiation, from either aging or underlying genetic risk. Luminal cancers are thought to arise from mature luminal cells and triple-negative breast cancers (TNBCs) are thought to arise from cKit^+^ progenitors^9–12^. We speculate that the differentiation defect observed in HR and AR older progenitors is a common point of convergence on the path of cancer susceptibility and initiation, and that they later diverge to produce the different subtypes of cancer (for example, TNBCs in *BRCA1*^mut37^, and luminal B in *BRCA2*^mut^ and *PALB2*^mut37,38^). It is reasonable to speculate that the epithelia and stroma carrying these different germline mutations generate microenvironments that favor growth of cancer cells of origin in a subtype-specific manner.

We focused mainly on understanding the changes in LEps because they showed the most pronounced age- and risk-dependent changes^7,39^. However, we also detected transcriptional changes in HR MEps consistent with loss of lineage fidelity, but the differences between HR and AR MEps were qualitatively less pronounced than the changes observed in LEps. This is consistent with our previous work in which age-dependent changes in MEps were difficult to detect^8,39^. We also showed a proportionate decrease in MEp populations in HR HMECs, another parallel to age-dependent changes^7^. The MEp-specific transcription factors p63 and TCF7 were shown to be perturbed in *BRCA1*^mut^ MEps and DCIS, causing loss of MEp lineage fidelity and decreased MEp proportions^40^. We observed a reduction in both these transcription factors in HR MEps that did not reach statistical significance. Our gene-overlap analyses showed overt enrichment of LEp-specific genes in HR MEps (inclusive of *BRCA1*, *BRCA2* and *PALB2* mutation carriers), which is consistent with the concept of perturbation of the MEp differentiation programs described by Ding et al.^40^. As the chronological age of MEps was shown to influence the expression of aging biomarkers in LEps^8^, an exciting possibility is that loss of fidelity in LEps is driven through risk-promoting microenvironments on apical surfaces of MEps.

The prospect that accelerated aging underlies high-risk biology merits further exploration because it provides new and untraveled avenues for conceptualizing cancer prevention strategies based on aging biology. The loss of lineage fidelity in LEps and the differentiation defect in cKit^+^ progenitors seem to appear in multiple different HR scenarios: for example, aging and carriers of *BRCA1*, *BRCA2* or *PALB2* mutations. However, the fact remains that different subtypes of cancers are expected to emerge from these four exemplar groups. Thus, it is possible that the differentiation bias and loss of lineage fidelity are just tempting red herrings, or that these intrinsic sources of risk are accompanied by unique microenvironmental changes that grant an adaptive advantage to cKit^+^ cells or their descendants with certain intrinsic states^41^. For example, albeit in vitro, Lim et al. showed that *BRCA1* epithelia seemed to have an advantage over normal in high-stress culture environments^9^. Enrichment of EMT, NF-κB signaling and JAK–STAT3 signatures in HR LEps and MEps may hint at early changes in the local microenvironment that create an adaptive niche and increase susceptibility to breast cancer initiation from a specific cell of origin. More detailed study of the progenitor differentiation defects and epithelial loss of fidelity in large DCIS and benign breast disease cohorts with longitudinal documentation would determine whether the same breast-aging biomarkers are universal emergent properties of breast tissue that is susceptible to cancer initiation, even in cancers that seem sporadic. The cKit^+^ progenitors and loss of lineage fidelity may be worthy targets for cancer prevention.

## Methods

### Human subjects.

The present study was approved by the Human Subjects Committee and the Institutional Review Board (IRB) at City of Hope. Women were consented in person and sequentially; all women signed a City of Hope IRB-approved consent before trial entry. Breast organoids from reduction mammoplasties were prepared at the Lawrence Berkeley National Laboratory (Berkeley, CA) with approved IRB for sample distribution and collection from specific locations. Women were eligible for the present study if they were undergoing a breast reduction or prophylactic mastectomy. Women were defined as HR if they had a germline mutation that increased their risk of breast cancer including *BRCA1*, *BRCA2* and *PALB2* (refs.^2,18^); women were defined as AR if they had a ≤12% lifetime risk of breast cancer by not possessing any predisposing germline mutation.

### General materials and reagents.

Reagents were used as received without modification.

### Cell culture.

Primary HMECs at passage 4 were grown and maintained in M87A medium as previously described^42,43^. HMECs from reduction mammoplasties were obtained from the HMEC Bank^44^. HMECs from prophylactic mastectomies and tissues contralateral or peripheral to tumors were obtained at City of Hope. *Mycoplasma* testing was performed on all cell strains before use.

### Immunofluorescence.

All formalin-fixed paraffin-embedded tissue sections were baked at 60 °C for 1–2 h. To deparaffinize the sections, slides were washed in xylene twice for 10 min each, 100% ethanol twice for 3 min each, 95% ethanol for 3 min and 70% ethanol for 3 min, and rinsed in Milli-Q water. Antigen retrieval was performed by incubating the slides with citric acid-based antigen unmasking solution (Vector Laboratories, catalog no. H-3300) at 90 °C for 8–10 min; slides were allowed to cool at room temperature until they reached 35 °C. Then, sections were washed with phosphate-buffered saline (PBS) for 5 min. All passage 4 samples were fixed using 1% paraformaldehyde for 10 min and washed three times in PBS for 10 min each.

Samples were incubated in blocking buffer (10% heat-inactivated goat serum in PBS + 0.5% Triton X-100) at 4 °C for a minimum of 24 h. Primary antibodies (anti-human KRT14, BioLegend, catalog no. 905301, 1:1,000; anti-human KRT19, BioLegend, catalog no. 628502, 1:1,000) were diluted into blocking buffer and incubated with the samples overnight. Samples were washed three times with PBS, stained with fluorophore-conjugated secondary antibodies (goat anti-rabbit immunoglobulin (Ig) G conjugated to Alexa Fluor-568 (Invitrogen, catalog no. A11011, 1:500); goat anti-mouse IgG2a conjugated to Alexa Fluor-647 (Invitrogen, catalog no. A21241, 1:500)) and Hoechst stain 33342 (Thomas Scientific, catalog no. C979U06, 1:200) for 2 h, and washed three times quickly with PBS at room temperature, then washed overnight in PBS at 4 °C. Samples were imaged the following day after mounting using Fluoromount-G mounting media (Electron Microscopy Sciences, catalog no.17984–25).

### Image acquisition.

Immunofluorescent images were taken using a Nikon Eclipse Ti2 with Nikon NIS-Elements viewer Software. Images of the pantomics tissue array sections of IDC and DCIS lesions were taken with a Zeiss LSM 800 confocal microscope with Airyscan running Zeiss Zen software v.3.1. Subsequent workup and image analysis were performed using ImageJ. Images were prepared for publication with Adobe Photoshop, where individual channels were merged and pseudo-colored.

### KRT19 and KRT14 quantification.

Immunofluorescent images were exported into monochannel TIFF images using Nikon NIS-Elements Viewer Software. The split-channel images were imported to ImageJ for analysis. An automated algorithm was used to quantify LEp cells positive for both KRT19 and KRT14. In this algorithm, the monochannel TIFF images representing KRT14 and KRT19 are converted into masks with automated thresholding (‘KRT19 mask’, representing LEp cells and ‘KRT14 mask’, representing MEp cells), and their area is measured. We then computed the area resulting from having both KRT14 and KRT19 (‘AND mask’) which indicates cells positive for both keratins, and the area resulting from having either KRT14 or KRT19 (‘OR mask’) which indicates all epithelial cells. Then we computed the ratio ‘AND mask’:’KRT19 mask’, reflecting LEps expressing both KRT19 and KRT14. The ratio ‘AND mask’:’OR mask’ reflects MEps and LEps that express both keratins. The ratio ‘AND mask’:’KRT14 mask’ reflects MEps expressing both KRT14 and KRT19.

### Flow cytometry.

Passage 4 mammary epithelial cells were stained with anti-human CD271 conjugated to PerCp-Cy5.5 (BioLegend, catalog no. 345112, 1:200) or CD271 conjugated to APC (BioLegend, catalog no. 345108, 1:200) or CD271 conjugated to FITC (BioLegend, catalog no. 345104, 1:200) and anti-human MUC1 conjugated to FITC (BD Biosciences, catalog no. 559774, 1:50) or anti-human CD133 conjugated to PE (BioLegend, catalog no. 372804, 1:200), and run through an Accuri C6 cytometer for flow cytometry analysis.

### Differentiation assay.

We enriched for cKit^+^ cells from passage 4 mammary epithelia using FACS when stained with cKit (CD117-PE; BioLegend, catalog no. 313206, 1:200). The cKit^+^ cells were plated and fixed with 1% paraformaldehyde at 2 or 7 d post-plating. To assess differentiation status, cKit^+^ cells were stained for differentiation markers KRT14 and KRT19. The immunofluorescent images obtained were run through a Cell Profiler pipeline for cell segmentation and quantification of KRT14 and KRT19 signals. Further quantification, plotting and statistical analysis was performed using R.

### RNA-seq.

Passage 4 HMECs were sorted into MEps and LEps using the markers described above. RNA extraction from the sorted cells was performed using a Quick-RNA Microprep kit with Zymo-Spin IC columns (Zymo Research, catalog no. R1050) and from TRIzol-homogenized adipose samples using a Direct-zol RNA Miniprep kit (Zymo Research, catalog no. R2051). Samples were then submitted to COH integrative genomics core for RNA-seq. RNA-seq libraries were prepared using KAPA RNA mRNA HyperPrep Kit (KAPABiosystems, catalog no. KR1352) according to the manufacturer’s protocol. Total RNA from each sample, 100 ng, was used for poly(A) RNA enrichment. Enriched mRNA underwent fragmentation and first-strand complementary DNA synthesis. The combined second cDNA synthesis with dUTP and A-tailing reaction generated the resulting double-stranded cDNA with dAMP on the 3′-ends. Barcoded adaptors were ligated to the double-stranded cDNA fragments. Ten cycles of PCR were performed to produce the final sequencing library. The libraries were validated using the Agilent Bioanalyzer DNA High Sensitivity Kit and quantified with Qubit. Sequencing was performed on an Illumina HiSeq 2500 with the single read mode of 51 cycles. Real-time analysis 2.2.38 software was used to process the image analysis. RNA-seq reads were trimmed to remove sequencing adapters using Trimmomatic^45^. The processed reads were mapped back to the human genome (hg19) using TOPHAT2 software^46^. HTSeq^47^ and RSeQC^48^ software packages were applied to generate the count matrices and strand information, respectively, using default parameters.

Differential gene expression analysis was performed in R using DESeq2 (ref.^49^). Gene set enrichment^50^, KEGG pathway and GO overrepresentation analyses were performed using clusterProfiler^51^. To compare our HR transcriptomic dataset with our previously sequenced AR younger and older samples, we used ComBat normalization and bridge samples. We defined MEp-specific genes as genes that are twofold upregulated in AR MEps compared with AR LEps with a Benjamini–Hochberg-adjusted *P*-value cutoff of 0.001. LEp-specific genes were established similarly. The aging signature in LEps was established by taking the upregulated genes in older LEps compared with younger LEps, with a Benjamini–Hochberg-adjusted *P*-value cutoff of 0.05. The aging signature in MEps was established similarly. The senescence gene signature was downloaded from the MSigDB. The gene-overlap analysis was performed using the GeneOverlap package^23^. The statistical significance of the overlap was assessed using *P* values derived from Fisher’s exact test^23^.

### Statistics and reproducibility.

We used power analysis through G*Power v.3.19.7 software to determine sample sizes. For two comparisons, we needed nine samples per group to attain a power of 0.95 and a significance level of 0.05, with a mean difference between groups of 23%. Before any statistical analysis, groups were compared for normal distribution and variance, when parametric assumptions were attained (groups were normally distributed and of equal variance), parametric tests were used such as independent sample Student’s *t*-test for two groups, one-way analysis of variance (ANOVA) with Tukey’s post-hoc test for three groups and more, or Pearson’s correlation for correlation analysis. Welch’s correction was used when equal variance was not attained. When the groups were not normally distributed, nonparametric tests were used such as the Mann–Whitney *U*-test for two groups, Kruskal–Wallis with Dunn’s post-hoc test for three groups and more, or Spearman’s correlation for correlation analysis. Each figure legend lists the exact statistical test used for each experiment and the frequency at which each experiment was repeated with similar results. Unless otherwise specified, all tests reported were two tailed. R v.4.0.3 software and GraphPad Prism v.8.3.0. software were used for statistical analysis. Significance was achieved when *P* < 0.05. Blinding was not part of our experimental design because we were using multiple biological and technical replicates and a semiautomated pipeline for analysis.

### Reporting Summary.

Further information on research design is available in the Nature Research Reporting Summary linked to this article.

## Extended Data

## Supplementary Material

Example of gating strategy

Supplementary Tables 1–12

Source Data Fig. 1

Source Data Fig. 3

## Data Availability

Request for further information and reagents should be directed to and will be fulfilled by the lead author. RNA-seq data have been deposited in the Gene Expression Omnibus database under accession no. GSE182338. Databases used in the present study include the MSigDB (https://www.gsea-msigdb.org/gsea/msigdb), the KEGG pathway database (https://www.genome.jp/kegg/pathway.html) and the GO database (http://geneontology.org). Data for reproducing the rest of the main figures in the paper have been provided as Supplementary Tables.
