# Supplementary material for: Evidence for accelerated aging in mammary epithelia of women carrying germline BRCA1 or BRCA2 mutations
Source: Nat Aging. Author manuscript; Available in PMC 2022 Mar 1. (PMC8849557; doi:10.1038/s43587-021-00104-9)
Supplement: Example of gating strategy [file NIHMS1772753-supplement-Example_of_gating_strategy.pdf]

nature portfolio

https://doi.org/10.1038/s43587-021-00104-9

Supplementary information

Evidence for accelerated aging in mammary epithelia of women carrying germline *BRCA1* or *BRCA2* mutations

In the format provided by the authors and unedited

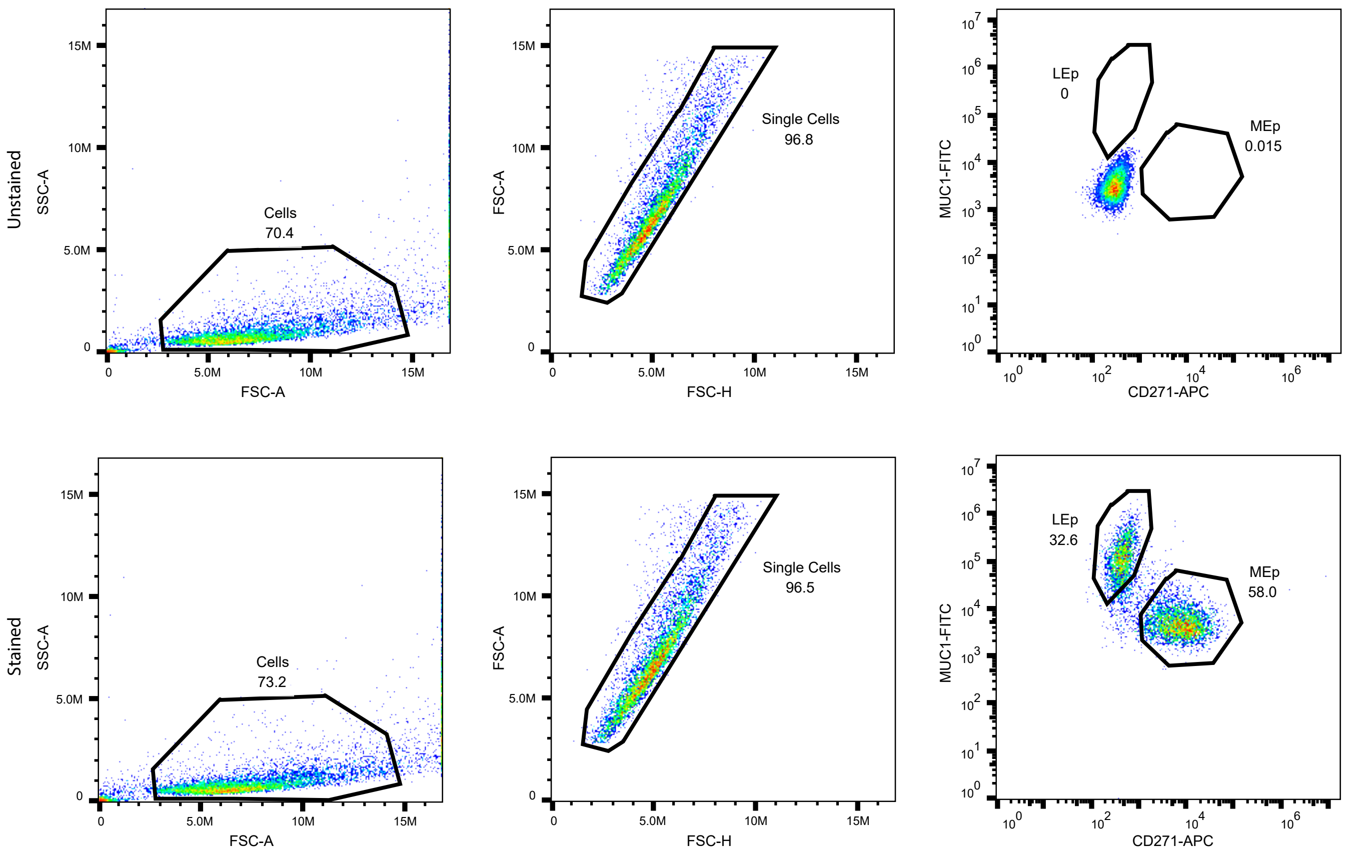
Unstained
Stained
SSC-A
FSC-A
FSC-H
Cells
70.4
73.2
Single Cells
96.8
96.5
LEp
0
32.6
MEp
0.015
58.0
MUC1-FITC
CD271-APC
15M
10M
5.0M
0
